# Chinese Herbal Medicine in the Treatment of Chronic Kidney Disease: A Narrative Review of Mechanisms and Therapeutic Potential

**DOI:** 10.5812/ijpr-165904

**Published:** 2025-11-04

**Authors:** Yao-Chou Tsai, Chung-Che Tsai, Ya-Hsuan Lin, Hsu-Hung Chang, Chan-Yen Kuo

**Affiliations:** 1 Division of Urology, Department of Surgery, Taipei Tzuchi Hospital, The Buddhist Tzu Chi Medical Foundation, New Taipei City, Taiwan; 2 Department of Research, Taipei Tzu Chi Hospital, The Buddhist Tzu Chi Medical Foundation, New Taipei City, Taiwan; 3 Department of Chinese Medicine, Taipei Tzu Chi Hospital, The Buddhist Tzu Chi Medical Foundation, New Taipei City, Taiwan; 4 Division of Nephrology, Department of Internal Medicine, Sijhih Cathay General Hospital, New Taipei City, Taiwan; 5 Institute of Oral Medicine and Material, College of Medicine, Tzu Chi University, Hualien, Taiwan

**Keywords:** Chronic Kidney Disease, Chinese Herbal Medicine, IgA Nephropathy

## Abstract

**Context:**

Chronic kidney disease (CKD) is a significant global health issue, characterized by its progressive nature and the limited effectiveness of conventional treatments. Chinese herbal medicine (CHM) has been used for centuries in East Asia to manage CKD by targeting key pathological processes. This review focuses on synthesizing evidence from various studies to explore the potential role of CHM in managing CKD, particularly through classical formulations, individual herbs, and their bioactive compounds with known nephroprotective effects.

**Evidence Acquisition:**

The review synthesizes evidence from preclinical studies, randomized controlled trials (RCTs), and meta-analyses to assess the effectiveness of CHM in CKD management. It highlights the use of classical CHM formulations like Liuwei Dihuang decoction, Zhenwu decoction, Shen-Qi-Wan (SQW), and Yu-Quan Wan (YQW), which are based on CHM theory and syndrome differentiation. Additionally, individual herbs such as *Astragalus membranaceus*, *Salvia miltiorrhiza*, *Rheum officinale*, *Tripterygium wilfordii *Hook F (TwHF), and *Cordyceps sinensis* are discussed for their nephroprotective bioactive compounds that provide multi-targeted effects, including anti-inflammatory, anti-fibrotic, antioxidant, and immunomodulatory actions.

**Results:**

Integration of CHM with standard therapy has shown promising results in improving renal function and reducing proteinuria, especially in patients with CKD and IgA nephropathy (IgAN). Evidence supports the multi-mechanistic actions of CHM, including the potential for its anti-inflammatory, anti-fibrotic, antioxidant, and immunomodulatory effects to positively impact CKD progression.

**Conclusions:**

Despite the growing body of evidence supporting the therapeutic value of CHM in CKD management, challenges still exist, such as the standardization of herbal preparations, ensuring safety, and gaining regulatory approval. For CHM to be more broadly integrated into evidence-based nephrology practice, further high-quality clinical research and harmonization of regulatory frameworks will be crucial. Overall, CHM offers a complementary approach to CKD treatment that aligns with classical principles and multi-targeted actions, contributing to a broader global strategy for CKD management.

## 1. Context

**Figure 1.**
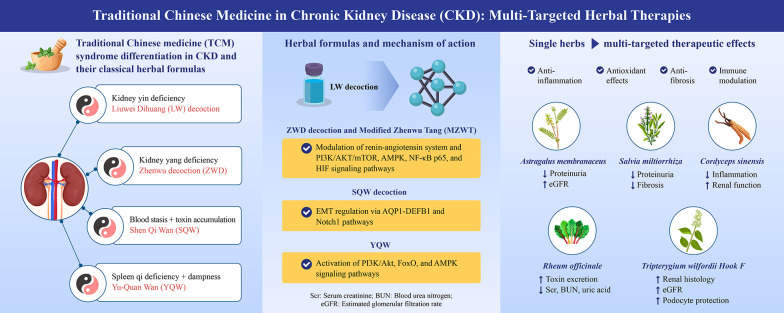
Summary of Chinese herbal medicine-based therapeutic strategies and mechanisms for chronic kidney disease. This graphical summary illustrates the CHM theoretical basis and syndrome differentiation in CKD management, categorizing major patterns such as kidney yin deficiency, kidney-yang deficiency, spleen qi deficiency with dampness, and blood stasis with toxin accumulation. Representative classical formulas, including LW, ZWD, MZWT, SQW, and YQW, are aligned with their associated CHM patterns and biological mechanisms. Arrows from each formula indicate the key pathways affected, such as PI3K/AKT/mTOR, AMPK, FoxO, NF-κB, and TGF-β/Smad, leading to anti-inflammatory, antioxidant, antifibrotic, and immunomodulatory effects. On the right side, major single herbs including *Astragalus membranaceus*, *Salvia miltiorrhiza*, *Rheum officinale*, *Tripterygium wilfordii* Hook F, and *Cordyceps sinensis* are shown to exert multi-targeted actions, with visual endpoints including reduced proteinuria, attenuation of renal fibrosis, and stabilization of renal function, including eGFR. The schematic integrates the content summarized in Tables 1 - 5, providing a holistic view of CHM interventions and their molecular and clinical relevance in CKD and related nephropathies, such as DKD and IgAN (Abbreviations: AMPK, AMP-activated protein kinase; CKD, chronic kidney disease; DKD, diabetic kidney disease; eGFR, estimated glomerular filtration rate; FoxO, forkhead box O; IgAN, immunoglobulin A nephropathy; LW, Liuwei Dihuang decoction; MZWT, modified Zhenwu Tang; NF-κB, nuclear factor-kappa B; PI3K/AKT/mTOR, phosphoinositide 3-kinase/protein kinase B/mammalian target of rapamycin; SQW, Shen-Qi-Wan; CHM, Chinese herbal medicine; TGF-β/Smad, transforming growth factor-beta/SMAD signaling pathway; YQW, Yu-Quan Wan; ZWD, Zhenwu decoction).

Chronic kidney disease (CKD) is a global public health challenge that affects approximately 10 - 15% of the adult population worldwide (1). The CKD is associated with high morbidity and mortality and a considerable socioeconomic burden. The CKD progression leads to impaired excretory, metabolic, and endocrine functions in the kidney, ultimately resulting in end-stage renal disease (ESRD) (2). Affecting more than 10% of the global population, CKD is associated with significant morbidity, mortality, and economic burden (3). The pathophysiology of CKD involves a complex interplay of hemodynamic, inflammatory, oxidative, and fibrotic processes that culminate in nephron loss and uremia (4). Despite advances in understanding disease mechanisms, current therapeutic strategies primarily aim to control blood pressure control, glycemic regulation in patients with diabetes, and the use of renin-angiotensin system inhibitors, aim to slow disease progression; however, they are not always effective in halting renal decline (5). Consequently, there is increasing interest in complementary and alternative treatments, particularly Chinese herbal medicine (CHM) , which provide multi-target therapeutic approaches (6). Conventional treatments for CKD include angiotensin-converting enzyme inhibitors (ACEIs), angiotensin II receptor blockers (ARBs), sodium-glucose co-transporter-2 (SGLT2) inhibitors, and phosphate binders (7, 8). While these agents provide partial protection against CKD progression, they are associated with limitations such as adverse effects, incomplete efficacy, and economic constraints (9, 10). As such, there is growing interest in complementary therapeutic strategies that may enhance current standards of care. Therefore, CHM has been used for centuries to manage kidney-related disorders (10). In modern research, CHM formulations and bioactive compounds have shown potential in attenuating renal inflammation, oxidative stress, and fibrosis, as well as improving renal function in both experimental and clinical settings (11). The integration of CHM with conventional medicine may offer a multifaceted approach to CKD treatment, especially in early or moderate disease stages. Several reviews have addressed the role of CHM in kidney disease; however, many of these focus on broad-spectrum CHM therapies without differentiating herbal interventions from acupuncture, Tuina, or other modalities (12, 13). Moreover, previous reviews have often lacked systematic comparison with conventional treatments or critical evaluation of clinical evidence quality. Therefore, an updated and focused review specifically addressing CHM’s potential mechanisms, clinical applications, and integration into evidence-based nephrology is warranted. The present review primarily aimed to comprehensively evaluate the therapeutic potential and underlying mechanisms of CHM in the management of CKD. By synthesizing findings from preclinical and clinical studies, this review seeks to clarify how CHM may contribute to renoprotection through mechanisms such as anti-inflammation, anti-oxidative stress, and anti-fibrosis. Additionally, the review intends to critically assess the advantages and limitations of CHM in comparison to conventional therapies and explore the rationale for integrative approaches. In doing so, this article also highlights gaps in existing literature and proposes directions for future research that may support the development of safe and effective CHM-based interventions for CKD. This article is a narrative review synthesizing evidence from preclinical studies, randomized controlled trials (RCTs), and meta-analyses retrieved from the PubMed, Scopus, and Web of Science databases. The review integrates findings from both clinical and experimental research to summarize therapeutic mechanisms, clinical efficacy, and emerging integrative strategies for the management of CKD and IgA nephropathy (IgAN) (Figure 1). The inclusion criteria were: (1) Original preclinical or clinical studies investigating CHM or herbal formulations in CKD or IgAN; (2) studies evaluating therapeutic mechanisms, efficacy, or safety; and (3) articles published in English. The exclusion criteria included: (1) Reviews, case reports, or non-original studies; (2) studies without clear outcome measures related to CKD; and (3) non-English publications. Data from the selected studies were synthesized narratively according to study type, intervention (classical formulations or individual herbs), and outcome. Although this is not a systematic review, a quality appraisal approach was applied by prioritizing RCTs, meta-analyses, and high-impact preclinical studies with reproducible methodologies. The strength and consistency of evidence were considered when interpreting findings. Classical formulations were selected based on their frequent documentation in traditional Chinese medicine theory and clinical usage for CKD management, including Liuwei Dihuang (LW) decoction, Zhenwu decoction (ZWD), Shen-Qi-Wan (SQW), and Yu-Quan Wan (YQW). Individual herbs, such as *Astragalus membranaceus*, *Salvia miltiorrhiza*, *Rheum officinale*, *Tripterygium wilfordii *Hook F (TwHF), and *Cordyceps sinensis*, were included due to their well-established nephroprotective mechanisms and consistent appearance in clinical studies and meta-analyses.

## 2. Chinese Herbal Medicine Perspectives on Chronic Kidney Disease: Syndrome Differentiation and Therapeutic Strategies

According to CHM theory, the kidney is the "root of life", responsible for storing essence (Jing), governing water metabolism, and maintaining bone and marrow health (14). The CKD is generally attributed to kidney deficiency and is often accompanied by dampness, heat, blood stasis, and toxin accumulation (15, 16). The CKD is frequently divided into different syndrome patterns, including kidney yin deficiency, kidney yang deficiency, blood stasis, turbid toxin retention, spleen qi deficiency, and dampness accumulation (16). Kidney yin deficiency is characterized by night sweats, a dry mouth, dizziness, and tinnitus (17). Kidney yang deficiency is characterized by cold limbs, fatigue, and edema (18). Spleen qi deficiency and dampness accumulation are common in patients with proteinuria, presenting with fatigue, poor appetite, and loose stools (19). Blood stasis and turbid toxin retention are often observed in the advanced stages and manifest as skin itching, dark complexion, and pain (16). The CHM therapy for CKD is based on syndrome differentiation (Bian Zheng Lun Zhi) and often combines herbal formulas that aim to tonify the kidney, invigorate the blood, promote diuresis, eliminate dampness, and clear heat (20).

## 3. Integrative Therapeutics for Kidney Disease: Chinese Herbal Medicine Formulas and Their Molecular Mechanisms

Liuwei Dihuang decoction is a CHM formula composed of six herbal ingredients: *Radix Rehmanniae* (Dihuang; the prepared root of *Rehmannia glutinosa*), *Rhizoma Dioscoreae* (Shanyao; the rhizome of *Dioscorea opposita), Fructus Corni* (Shanzhuyu; the fruit of *Cornus officinalis*), *Cortex Moutan Radicis* (Mudanpi; the root bark of *Paeonia suffruticosa*), *Rhizoma Alismatis* (Zexie; the rhizome of *Alisma plantago-aquatica*), and *Poria* (Fuling; the sclerotia of *Poria cocos*) and is widely used in patients with CKD with yin deficiency symptoms (21). It has demonstrated antioxidant, anti-inflammatory, and anti-fibrotic effects in animal models of renal injury (22). The ZWD is a well-known classical CHM prescription used in cases of kidney yang deficiency with water retention (23). Previous studies have suggested that ZWD exerts a protective effect against renal damage in diabetic nephropathy (DN) by modulating the renal renin-angiotensin system and preserving podocyte integrity, thereby mitigating proteinuria and structural kidney damage (24). In contrast, Liu et al. demonstrated that ZWD induces mitophagy, thereby protecting mitochondrial function in chronic glomerulonephritis (CGN). This protective effect is mediated by modulating the phosphoinositide 3-kinase (PI3K)/protein kinase B (AKT or PBK)/mammalian target of rapamycin (mTOR) and 5' adenosine monophosphate-activated protein kinase (AMPK) signaling pathways (25). Another study showed that Zhenwu Tang (ZWT) exerts substantial protective effects against adriamycin-induced nephrotic syndrome (NS) in rats. The ZWT treatment effectively improved renal function, reduced proteinuria and serum lipid levels, and preserved the glomerular structure. Mechanistically, ZWT attenuated inflammation by downregulating NF-κB p65 expression and upregulating IκB, modulated cytokine profiles by decreasing IL-8 and increasing IL-4 levels, and enhanced antioxidant defenses through reduced malondialdehyde levels and increased superoxide dismutase activity, suggesting that ZWT mitigates renal injury by modulating inflammatory and oxidative stress pathways, supporting its therapeutic potential as a CHM for managing CKDs, such as NS (26). Similar results have indicated a renoprotective effect of ZWD on renal fibrosis by regulating oxidative damage and energy metabolism imbalance (27). Modified Zhenwu Tang (MZWT) effectively attenuates renal dysfunction by targeting these pathogenic mechanisms. Both in vivo and in vitro findings demonstrated that MZWT reduced oxidative stress, inhibited hypoxia-inducible factor (HIF) signaling pathway activation, suppressed epithelial-mesenchymal transition, and decreased inflammatory cytokine and collagen production in renal proximal tubular epithelial cells (28). Therefore, both LW and ZWD demonstrate substantial therapeutic potential in CKD through their multifaceted mechanisms of action. The LW has shown promising effects in animal models by alleviating oxidative stress, inflammation, and fibrosis, making it a valuable treatment option for patients with CKD and yin deficiency symptoms. The ZWD, known for its protective role in kidney-yang deficiency, has been found to mitigate renal damage by modulating key signaling pathways, including the renin-angiotensin system and the PI3K/AKT/mTOR/AMPK pathways. Furthermore, MZWT effectively reduced oxidative stress and inflammatory responses while protecting renal function by targeting energy metabolism and fibrosis. Collectively, these findings underscore the therapeutic potential of these CHM formulations in managing renal dysfunction and enhancing kidney health in patients with CKD (Table 1). 

**Table 1.** Summary of Chinese Herbal Medicine Decoctions for Chronic Kidney Disease and Their Mechanisms

Formula	Composition	Indication (CHM Pattern)	Mechanisms of Action	Experimental Evidence	References
**LW**	*Radix Rehmanniae* (Dihuang), *Rhizoma Dioscoreae* (Shanyao), *Fructus Corni* (Shanzhuyu), *Cortex Moutan Radicis* (Mudanpi), *Rhizoma Alismatis* (Zexie), *Poria* (Fuling)	Kidney yin deficiency	Antioxidant, anti-inflammatory, anti-fibrotic effects	Animal models of renal injury	(21, 22)
**ZWD**	*Poria*, Aconiti Lateralis Radix Praeparata, white Atractylodes, ginger, and prepared Licorice	Kidney-yang deficiency with water retention	Modulates renin-angiotensin system; Preserves podocyte integrity; Induces mitophagy; Regulates PI3K/AKT/mTOR and AMPK pathways	DN, CGN models	(23, 25)
**ZWT**	Same base as ZWD	NS model	Reduces proteinuria and serum lipids; Downregulates NF-κB p65, upregulates IκB; Decreases IL-8, increases IL-4; Enhances SOD activity, lowers MDA	Adriamycin-induced NS in rats	(26)
**ZWD**	-	CKD with renal fibrosis	Regulates oxidative damage; Corrects energy metabolism imbalance	Animal models of renal fibrosis	(27)
**MZWT**	Modified from ZWD/ZWT	CKD with renal fibrosis	Reduces oxidative stress; Inhibits HIF signaling; Suppresses EMT; Decreases inflammatory cytokines and collagen production	in vivo and in vitro (renal epithelial cells)	(28)

Abbreviations: AKT, protein kinase B; AMPK, AMP-activated protein kinase; CGN, chronic glomerulonephritis; CHM, Chinese herbal medicine; CKD, chronic kidney disease; EMT, epithelial-mesenchymal transition; HIF, hypoxia-inducible factor; IL, interleukin; LW, Liuwei Dihuang decoction; MDA, malondialdehyde; MZWT, modified Zhenwu Tang; mTOR, mammalian target of rapamycin; NF-κB, nuclear factor-kappa B; NS, nephrotic syndrome; PI3K, phosphoinositide 3-kinase; SOD, superoxide dismutase; ZWD, Zhenwu decoction; ZWT, Zhenwu Tang; DN, diabetic neurophatic.

The SQW is a CHM formula used to invigorate ShenYangXu (SYX; kidney-yang deficiency syndrome), with a history of use spanning thousands of years in Asia. It was first documented in the "Synopsis of the Golden Chamber" and is composed of *Radix Rehmanniae Preparata*, *Fructus Macrocarpii*, *Rhizoma Dioscoreae Oppositae*, *Rhizoma Alismatis*, *Poria*, *Cortex Moutan Radicis*, *Radix Aconiti Lateralis Preparata*, and *Ramulus Cinnamomi*. The SQW demonstrated its protective effects on glomerular structure and function (29). Liu et al. demonstrated that SQW substantially reduced renal collagen deposition, decreased serum inflammatory cytokine levels, and improved renal function. β-defensin 1 (DEFB1) has emerged as a key therapeutic target, with SQW modulating its expression via aquaporin 1 (AQP1). Notably, the renal protective effects of SQW were partially diminished after AQP1 gene knockout, further confirming the importance of the AQP1-DEFB1 pathway in its action (30). Similar results indicated that SQW attenuates renal interstitial fibrosis (RIF) by inhibiting epithelial-to-mesenchymal transition (EMT), primarily through AQP1 upregulation. Both in vivo and in vitro models have demonstrated that SQW reduces renal injury and fibrosis, increases E-cadherin and AQP1 expression, and suppresses mesenchymal markers, such as vimentin and α-SMA. Notably, AQP1 knockdown reversed these protective effects, promoting EMT and abolishing the therapeutic benefits of SQW (31). Another study demonstrated that SQW exerted a protective effect against RIF by modulating the EMT and Notch1 signaling pathway. The SQW-containing serum enhanced the viability of transforming growth factor-beta (TGF-β)-induced HK-2 cells and mitigated the expression of fibrotic markers, including fibronectin, α-SMA, vimentin, N-cadherin, and collagen I, while promoting the collagen II and E-cadherin levels. Additionally, SQW modulated the upregulation of Notch1 and associated proteins triggered by TGF-β, and Notch1 knockdown further amplified its effects (32). Collectively, these findings underscore the multifaceted renoprotective effects of SQW and support its continued exploration as a promising therapeutic strategy for treating CKD.

The YQW is a modern polyherbal formulation derived from traditional Yuquan powder, recorded in volume II (diabetes) of the ancient text Zhongfutang Public Selection Recipe. It comprises six herbal components: *Pueraria lobata* (Willd.) Ohwi (Gegen), *Rehmannia glutinosa* (Gaertn.) DC. (Dihuang), *Ophiopogon japonicus* (Thunb.) Ker Gawl. (Maidong), *Trichosanthes kirilowii* Maxim./*Trichosanthes rosthornii* Harms (Tianhuafen), *Schisandra chinensis* (Turcz.) Baill. (Wuweizi), and *Glycyrrhiza* species, including *Glycyrrhiza uralensis* Fisch., G. *inflata* Batalin, and *G. glabra* L. (Gancao). Among these, Gegen is a monarch herb that promotes fluid production, relieves thirst, and reduces internal heat. It is traditionally associated with the lung and stomach meridians (33). Additionally, the results also demonstrate that YQW exerts therapeutic effects in type 2 diabetes mellitus by modulating gene expression and targeting key signaling pathways, such as PI3K/Akt, Forkhead Box O (FoxO), and AMPK. The active constituents, including puerarin, daidzein, and glycyrrhetinic acid, appear to regulate critical proteins, such as FoxO3, IL10, Ppargc1a, and FoxO1, thereby alleviating inflammation, correcting lipid metabolism disorders, and protecting liver and kidney function. Moreover, YQW reversed the expression of five key genes, Kit, Ppard, Ppara, Fabp4, and Tymp, highlighting its potential to mitigate oxidative stress and metabolic dysfunction under diabetic conditions (33). The YQW traditionally treats polydipsia by clearing internal heat, promoting fluid production, relieving thirst, and moistening dryness, which are symptoms commonly associated with hyperglycemia (34). It also nourishes yin and generates fluids that are often used in DN (35). Therefore, these results support the clinical relevance of YQW in managing metabolic dysfunction and oxidative stress, especially in DN, while bridging the traditional herbal theory with modern molecular insights (Table 2). 

**Table 2.** Summary of Shen-Qi-Wan and Yu-Quan Wan for Treating Chronic Kidney Disease and Diabetic Nephropathy

Formula	Composition	Indication (CHM Pattern)	Mechanisms of Action	Experimental Evidence	References
**SQW**	*Radix Rehmanniae Preparata*, *Fructus Macrocarpii*, *Rhizoma Dioscoreae Oppositae*, *Rhizoma Alismatis*, *Poria*, *Cortex Moutan Radicis*, *Radix Aconiti Lateralis Preparata*, *Ramulus Cinnamomi*	Kidney-yang deficiency (SYX)	Protects glomerular structure and function; Reduces collagen deposition and inflammation; Modulates AQP1-DEFB1 axis; Inhibits EMT and renal fibrosis via AQP1; Modulates Notch1 signaling pathway	in vivo and in vitro models of renal injury and fibrosis	(29-32)
**YQW**	*Pueraria lobata* (Gegen), *Rehmannia glutinosa* (Dihuang), *Ophiopogon japonicus* (Maidong), *Trichosanthes kirilowii/rosthornii* (Tianhuafen), *Schisandra chinensis* (Wuweizi), *Glycyrrhiza* spp. (Gancao)	Yin deficiency with internal heat; diabetic nephropathy	PI3K/Akt, FoxO, and AMPK pathways; Regulates lipid metabolism and oxidative stress; Modulates expression of FoxO1, FoxO3, Ppargc1a, IL-10, and others; Reverses expression of genes involved in metabolism (e.g., Kit, Ppard, Ppara)	Systems pharmacology and transcriptomics in T2DM models	(33-35)

Abbreviations: AKT, protein kinase B; AMPK, AMP-activated protein kinase; CHM, Chinese herbal medicine; EMT, epithelial-mesenchymal transition; IL, interleukin; PI3K, phosphoinositide 3-kinase; SQW, Shen-Qi-Wan; SYX, ShenYangXu; T2DM, type 2 diabetes mellitus; YQW, Yu-Quan Wan.

## 4. Chinese Herbal Medicines in Treating Chronic Kidney Disease: Therapeutic Mechanisms, Clinical Evidence, and Modern Application

Many CHMs contain active compounds that act on multiple pathways relevant to CKD pathophysiology (36). *Astragalus membranaceus* (Huang Qi) is a traditional CHM with a history of clinical use spanning thousands of years. Because of its ability to strengthen the spleen and replenish vital energy (qi), it is commonly prescribed in CHM to treat conditions associated with qi deficiency (37). In patients with type 2 diabetes, stage 2 to 3 CKD, and macroalbuminuria, the addition of astragalus to standard care for 48 weeks further helped stabilize kidney function (38). Liu et al. provided compelling evidence that total flavonoids of astragalus (TFA) offer protective effects against diabetic kidney disease (DKD) by preserving the integrity of the glomerular filtration barrier. The TFA significantly reduced proteinuria and improved kidney function in diabetic mice, while also attenuating oxidative stress and inflammation in glomerular endothelial cells. Mechanistically, TFA exerts its renoprotective effects by enhancing the expression of tight junction proteins and core components of the endothelial glycocalyx (syndecan-1 and glypican-1), improving mitochondrial function, and modulating the PI3K/AKT signaling pathway (39). This retrospective, self-controlled case series demonstrated a statistically significant improvement in estimated glomerular filtration rate (eGFR) among patients with mild-to-moderate CKD after using astragalus-containing preparations (40). This review highlights the potential of *A. membranaceus* (Huangqi) as a therapeutic agent for treating diabetic nephropathy (DN), emphasizing its role in modulating immune responses, particularly through macrophage inducible nitric oxide synthase (iNOS) activity regulation. Furthermore, the dual regulatory action of Huangqi, suppressing iNOS expression in early inflammatory stages and enhancing it in fibrotic stages, suggests a context-dependent immunomodulatory effect that may help prevent DN progression (41). A systematic review and meta-analysis evaluated the efficacy of Huangqi injection, an extract of *A. membranaceus*, combined with antihypertensive drugs, for treating hypertensive nephropathy. The results indicated that combination therapy significantly improved several clinical parameters compared to antihypertensive drugs alone, including reductions in 24-h urinary total protein, microalbuminuria, serum creatinine (SCR), systolic and diastolic blood pressure, cystatin C, and blood urea nitrogen (BUN) (42). Collectively, these findings highlight *A. membranaceus* as a promising adjunctive therapy to conventional treatments for CKD and DKD, warranting further rigorous large-scale clinical trials to validate its efficacy and mechanistic pathways.

*Salvia miltiorrhiza* Bunge, commonly referred to as Danshen in Chinese and Dansam in Korea, is also widely known as Chinese sage or red sage (43). A systematic review and meta-analysis showed that *S. miltiorrhiza* may offer potential benefits as a complementary therapy for patients with CKD, including improved kidney function, reduced proteinuria, slowed CKD progression, and alleviated various related complications (44). Tanshinone IIA, a major active compound in *S. miltiorrhiza*, has a promising therapeutic potential for CKD. In a 5/6 nephrectomy rat model, chronic oral Tanshinone IIA administration significantly improved renal function by reducing SCR, angiotensin II, TGF-β1, and collagen IV levels and decreasing urinary protein excretion (45). Hsu et al. demonstrated that the ethyl acetate (EtOAc) extract of *S. miltiorrhiza* exhibited significant renoprotective effects against diabetic nephropathy both in vitro and in vivo. The EtOAc layer effectively activates PPAR-α and PPAR-γ signaling pathways, suppresses pro-inflammatory and pro-fibrotic markers such as IL-1β, TGF-β1, and fibronectin, and restores PPAR-γ expression in high-glucose–stimulated mesangial cells. Furthermore, it inhibits myofibroblast activation and ameliorates renal hypertrophy, proteinuria, and fibrosis in diabetic mouse models (46). Collectively, these findings support the potential of *S. miltiorrhiza* as a promising adjunct therapy for managing kidney diseases, warranting further clinical investigations to confirm its efficacy and safety in humans.

*Rheum officinale* (Da Huang), a medicinal herb widely used in China to treat CKD, has been reported to have various pharmacological properties that may delay disease progression (47). A systematic review and meta-analysis demonstrated that rhubarb (*R. officinale*) and rhubarb-containing CHMs formulas offer promising therapeutic benefits in patients with chronic renal failure (CRF). The pooled results from 34 clinical studies involving 2,786 patients showed that rhubarb significantly reduced SCR, BUN, and uric acid (UA) levels, while increasing the creatinine clearance rate (CCR) and improving overall clinical symptom efficacy (48). Traditionally, *R. officinale *has been used to eliminate turbidity and detoxify the body. Recent studies have revealed its nephroprotective mechanisms, primarily by enhancing uremic toxin excretion via the colon and reducing RIF (49). A population-based nested case-control study provided compelling evidence that the integration of CHMs, including Da Huang, into routine care for patients with rheumatoid arthritis (RA) was associated with a significantly reduced risk of developing CKD (50). *Rheum palmatum* (Chinese rhubarb, Da Huang) has also been used as a botanical agent to relieve CRF (51). Overall, these findings highlight the potential of *R. officinale* as an adjunct therapy to slow CKD progression and improve patient outcomes, warranting further research and clinical validation.

The TwHF, an herbal medicinal extract, has been widely used by Chinese nephrologists for nearly four decades to treat glomerulonephritis. Its therapeutic effects include anti-inflammatory, anti-proliferative, and podocyte-protective effects, which are primarily attributed to its active triptolide monomer (52). Cheng et al. extended their potential therapeutic applications to DKD. This single-center cohort study investigated the effects of TwHF on renal outcomes in patients with type 2 DKD and severe proteinuria (53). A comprehensive review and meta-analysis of 103 RCTs suggested that TwHF may exert substantial nephroprotective effects against various kidney disorders. The TwHF treatment was associated with reduced 24-h proteinuria, serum creatinine, and BUN levels, as well as improved overall treatment efficacy and a lower incidence of adverse reactions than placebo, conventional Western medicine, and other immunosuppressive therapies (54). Zhu et al. have reported that *Tripterygium*-based preparations may exert protective effects on kidney function (55). A previous study systematically investigated the therapeutic mechanisms of TwHF-*T*. *kirilowii* Maxim decoction (LTD) in treating DKD using network pharmacology, molecular docking, and in vitro experiments. The findings revealed that LTD exerts renoprotective effects by targeting multiple signaling pathways involved in DKD pathogenesis, particularly by downregulating the expression of key inflammatory and stress-related proteins, such as PTGS2, NF-κB, JNK, and AKT (56). Triptolide, a bioactive compound derived from TwHF, has been shown to exert protective effects on podocytes in a diabetic nephropathy mouse model through Nrf2/HO-1 signaling pathway activation and (NOD-, LRR-, and pyrin domain-containing protein 3) NLRP3 inflammasome pathway suppression (57). Overall, these findings support the therapeutic potential of TwHF and its derivatives for treating kidney diseases.

*Cordyceps sinensis* (*Cordyceps*, Dong Chong Xia Cao), also known as the Chinese caterpillar fungus, is one of the most widely used traditional CHMs for managing CKD (58) and DKD (59, 60). In addition to conventional therapies that target hyperglycemia, hypertension, and proteinuria, *C. sinensis* offers a multifaceted approach by exerting nephroprotective, anti-inflammatory, antioxidant, anti-fibrotic, and immunomodulatory effects. Clinical evidence and traditional usage support its role in tonifying the lungs and kidneys, aligning with its therapeutic application in DKD (61). Preclinical studies using diverse animal models — such as unilateral ureteral obstruction, DN, 5/6 nephrectomy, and aristolochic acid nephropathy-demonstrate that *C. sinensis* alleviates renal fibrosis primarily through inhibition of EMT, modulation of TGF-β1/Smad, mitogen-MAPK, NF-κB, Sirtuin 1 (SIRT1), and NLRP3 inflammasome pathways, and enhancement of anti-inflammatory responses. In vitro studies further supported its direct effects on renal tubular epithelial cells, fibroblasts, and macrophages. Moreover, clinical evidence suggests that medicines derived from *C. sinensis* mycelia, such as Jinshuibao and Bailing capsules, improve renal function and reduce inflammation and extracellular matrix deposition in patients with CKD (62). This comprehensive meta-analysis and network pharmacology study demonstrated that Bailing capsules derived from *C. sinensis* have substantial potential as an adjunct therapy for CKD. Clinical trial data reveal that Bailing capsules, when combined with standard treatments, effectively reduce SCR, BUN, and 24-h urinary protein (24-hUP), while lowering inflammatory markers such as tumor necrosis factor-alpha (TNF-α), IL-6, and hs-CRP. Network pharmacology analysis further elucidates the multi-component, multi-target, and multi-pathway mechanisms underlying these effects, identifying key signaling pathways, including NF-κB, TGF-β1/Smad, PPAR, and AGE-RAGE, which are associated with immune regulation, inflammation, oxidative stress, and fibrosis (63). These findings highlight the therapeutic potential of *C. sinensis* and its derivatives as effective adjunct treatments for kidney diseases (Table 3). 

**Table 3.** Summary of Key Traditional Chinese Herbal Medicines Used for Treating Chronic Kidney Disease and Diabetic Kidney Disease

Herb	Traditional Use/CHM Function	Active Constituents	Mechanisms of Action	Therapeutic Effects	References
***Astragalus membranaceus*** ** (Huang Qi)**	Tonifies qi, supports spleen and kidney	Total flavonoids, polysaccharides	Preserves glomerular filtration barrier; Enhances tight junctions and glycocalyx; Activates PI3K/AKT; Modulates iNOS and mitochondrial function	Stabilizes renal function in T2DM with CKD; Reduces proteinuria and inflammation; Improves eGFR and blood pressure in CKD	(36-42)
***Salvia miltiorrhiza*** ** (Danshen)**	Invigorates blood, clears heat	Tanshinone IIA, EtOAc extract	Activates PPAR-α/γ pathways; Suppresses IL-1β, TGF-β1, fibronectin; Reduces collagen IV and Ang II; Inhibits myofibroblast activation	Reduces proteinuria; Improves renal function in CKD/DKD; Attenuates renal fibrosis and inflammation	(43-46)
***Rheum officinale*** ** (Da Huang)**	Purges accumulation, detoxifies	Anthraquinones, rhein	Promotes uremic toxin excretion via the colon; Reduces RIF; Improves creatinine clearance	Decreases SCR, BUN, UA; Enhances CCR; Reduces CKD risk in RA patients	(47-51)
**TwHF**	Dispels wind and dampness; relieve swelling	Triptolide	Inhibits NF-κB, JNK, AKT; Activates Nrf2/HO-1; Suppresses NLRP3 inflammasome; Protects podocytes	Reduces proteinuria and inflammatory markers; Improves renal histology and eGFR; Beneficial in glomerulonephritis and DKD	(52-57)
***Cordyceps sinensis*** ** (Dong Chong Xia Cao)**	Tonifies lungs and kidneys, boosts essence	Cordycepin, polysaccharides	Inhibits EMT and renal fibrosis; Modulates TGF-β1/Smad, MAPK, NF-κB, SIRT1, AGE-RAGE; Enhances anti-inflammatory responses	Improves renal function; Reduces inflammation and ECM deposition; Effective in CKD/DKD patients via Bailing and Jinshuibao capsules	(58-63)

Abbreviations: AGE-RAGE, advanced glycation end product-receptor for advanced glycation end products; AKT, protein kinase B; BUN, blood urea nitrogen; CCR, creatine clearance rate; CHM, Chinese herbal medicine; CKD, chronic kidney disease; DKD, diabetic kidney disease; eGFR, estimated glomerular filtration rate; ECM, extracellular matrix; EMT, epithelial-mesenchymal transition; IL, interleukin; iNOS, inducible nitric oxide synthase; JNK, c-jun N-terminal kinase; MAPK, mitogen-activated protein kinase; NF-κB, nuclear factor-kappa B; NLRP3, nod-like receptor family pyrin domain-containing 3; PI3K, phosphoinositide 3-kinase; RA, rheumatoid arthritis; SCR, serum creatinine; SIRT1, sirtuin 1; T2DM, type 2 diabetes mellitus; TGF, transforming growth factor; TwHF,* Tripterygium wilfordii* Hook F; UA, uric acid.

## 5. Therapeutic Potential of Chinese Herbal Medicines in Chronic Kidney Disease and IgA Nephropathy: Clinical Evidence, Mechanisms, and Emerging Integrative Strategies

Several well-designed RCTs and meta-analyses have demonstrated that the use of CHMs as adjuncts to conventional therapies may provide significant benefits for patients with CKD (64). Goto et al. reported that *Astragalus *root exerts renoprotective effects through anti-inflammatory and antifibrotic mechanisms, as well as by improving renal function (65). Notably, *Astragalus* injection demonstrated superior therapeutic effects in patients with DN, showing notable renal protective benefits, such as improvements in BUN, SCR, CCR, and urinary protein levels, as well as enhanced systemic conditions, as evidenced by increased serum albumin levels compared to the control group (66). Similar results suggest that *A. membranaceus* may help stabilize the eGFR and delay the need for renal replacement therapy in patients with progressive stage 4 CKD (67). A comprehensive meta-analysis evaluated the efficacy of *A. membranaceus* as an adjunct therapy in patients with moderate-to-high-risk idiopathic membranous nephropathy. The results indicated that combining *A. membranaceus* with standard treatments substantially improved clinical outcomes, including higher complete and partial remission rates, reduced proteinuria, and increased serum albumin levels (68). A previous study demonstrated that *Astragalus*-based Eefooton (EFT) significantly reduced proteinuria and improved kidney function (69). In contrast, Lu et al. speculated that this provides compelling clinical and experimental evidence supporting the therapeutic potential of EFT, an *Astragalus*-based CHMs formulation, as an adjunctive treatment for CKD, particularly in stages 3 - 5. Over a 6-month treatment period, EFT significantly improved renal function, as indicated by an increased eGFR, without inducing notable adverse events. In vitro studies further revealed that EFT enhanced renal tubular epithelial cell viability, improved clonogenicity, and suppressed fibrosis-related protein expression, primarily by inhibiting the poly (ADP-ribose) polymerase 1 (PARP-1) signaling pathway, suggesting that EFT may attenuate fibrosis and cellular injury in CKD, thus offering a complementary strategy to conventional therapies (70). Collectively, these findings underscore the value of *Astragalus *and its formulations as effective adjunct therapies for CKD (Table 4). 

**Table 4.** Summary of Evidence for *Astragalus*-Based Therapies in Treating Chronic Kidney Disease

Intervention	Study Type	Mechanisms of Action	Therapeutic Effects	Patient Population/Model	References
***Astragalus *** **root**	Clinical observation	Anti-inflammatory, anti-fibrotic	Improves renal function	CKD patients	(65)
***Astragalus*** ** injection**	RCT	Not specified in detail	Reduces BUN, SCR, urinary protein; increases serum albumin	DN patients	(66)
***Astragalus*** ***membranaceus*** ** (oral)**	Clinical study	Stabilizes renal function	Delays eGFR decline and renal replacement therapy	Progressive stage 4 CKD	(67)
***Astragalus*** **+standard therapy**	Meta-analysis	Not specified	Increases complete/partial remission; reduces proteinuria; increases serum albumin	Moderate to high-risk IMN	(68)
**EFT, ** ***Astragalus*** **-based CHM**	Clinical and in vitro studies	Inhibits PARP-1 pathway; reduces fibrosis	Improves eGFR, reduces fibrosis-related proteins, enhances cell viability	CKD stage 3 - 5 patients and renal epithelial cell models	(69, 70)

Abbreviations: BUN, blood urea nitrogen; CHM, Chinese herbal medicine; CKD, chronic kidney disease; DN, diabetic nephropathy; eGFR, estimated glomerular filtration rate; EFT, Eefooton; IMN, idiopathic membranous nephropathy; PARP, poly (ADP-ribose) polymerase; RCT, randomized controlled trial; SCR, serum creatinine.

Immunoglobulin A nephropathy (IgAN) is the most common primary glomerular disease globally and is associated with poor outcomes including CKD progression and kidney failure (71). Recently, strategies for IgAN treatment have aimed to reduce nephron loss, inflammation, fibrosis, and pathogenic IgA production, offering hope for more effective, multitarget interventions (72). The CHM has demonstrated effectiveness in managing IgAN and holds promise as a potential alternative therapeutic approach by strengthening the spleen, nourishing the kidneys, eliminating dampness and clearing turbidity, promoting blood circulation, resolving stasis, and unblocking channels while harmonizing collaterals (73). A systematic review and meta-analysis of preclinical animal studies demonstrated that CHM interventions significantly improved key renal function and pathological markers, including 24-h urine protein, urinary red blood cells, serum creatinine, BUN, TNF-α, TGF-β1, the matrix metalloproteinase-9/tissue inhibitor of metalloproteinases (TIMP)-1 ratio, and nephrin mRNA expression. However, no significant improvements were observed in serum albumin and IL-6 levels (74). The CHM-WINE study is a prospective, double-blind, randomized controlled trial designed to assess the efficacy and safety of the Yi-Qi-Qing-Jie formula (YQF), a CHM herbal compound, in combination with standard immunosuppressive therapy for high-risk nephropathy (IgAN) patients. These findings are expected to provide robust clinical evidence supporting the integration of CHM with conventional treatments, potentially offering a novel therapeutic strategy for managing high-risk IgAN patients (75). Ma et al. reported that combining CHM with ACEIs or ARBs offers superior therapeutic benefits compared to monotherapy with either CHM or ACEIs/ARBs alone for the treatment of IgAN. Specifically, combination therapies are more effective in reducing proteinuria and improving renal function markers, suggesting a synergistic effect (76). Nephrokeli (NPKL), a traditional Chinese herbal formula, alleviates kidney injury in a rat model of IgAN by modulating the sphingosine-1-phosphate (S1P) signaling pathway (22). In contrast, CHM can potentially improve fatigue, appetite, anemia, and the overall quality of life in dialysis patients (77). Overall, CHM represents a valuable complementary approach for IgAN management, warranting further clinical validation and integration into evidence-based treatment protocols (Table 5). 

**Table 5.** Summary of Chinese Herbal Medicine-Based Strategies for IgA Nephropathy Management

Intervention/Formula	Study Type	Mechanisms/Effects	Therapeutic Outcomes	Target Population/Model	References
**General CHM (multi-formula)**	Systematic review and meta-analysis (preclinical)	↓ Inflammation (TNF-α, TGF-β1), ↓ 24 h urine protein, ↑ nephrin mRNA, modulates MMP-9/TIMP-1	Improved renal function and pathology; no effect on IL-6 or serum albumin	Animal models of IgAN	(74)
**Yi-Qi-Qing-Jie formula (YQF)**	Prospective, double-blind RCT (CHM-WINE)	Integrates with immunosuppressive therapy	Expected to enhance efficacy and safety in high-risk IgAN	High-risk IgAN patients	(75)
**CHM+ACEI/ARB combination**	Comparative clinical study	Synergistic modulation of RAAS and inflammation	↓ Proteinuria, ↑ renal function vs. monotherapy	IgAN patients	(76)
**NPKL**	Preclinical (rat model)	Modulates S1P signaling pathway	Ameliorates kidney injury in IgAN	IgAN rat model	(22)
**General CHM use in dialysis patients**	Clinical observation	Symptom relief, Qi and blood support	↑ Appetite, ↓ fatigue, ↑ quality of life, improved anemia	Dialysis patients (CKD stage 5)	(77)

Abbreviations: ACEI, angiotensin-converting enzyme inhibitors; ARB, angiotensin II receptor blockers; CHM, Chinese herbal medicine; CKD, chronic kidney disease; IgAN, IgA nephropathy; IL, interleukin; MMP, matrix metalloproteinase; RAAS, renin-angiotensin-aldosterone system; TGF, transforming growth factor; TIMP, tissue inhibitor of metalloproteinases; TNF, tumor necrosis factor; NPKL, Nephrokeli.

## 6. Challenges to the Integration of Chinese Herbal Medicine in Chronic Kidney Disease Management

Despite the growing body of evidence supporting the efficacy of CHM and herbal formulations in CKD management, several challenges remain before their widespread integration into mainstream nephrology can be fully realized. First, there are limited high-quality clinical trials; many studies on CHM consist of preclinical data, or small-scale clinical trials that have methodological limitations, such as a lack of blinding, randomization, or adequate control groups. Robust multicenter RCTs with standardized protocols are essential to validate the safety, efficacy, and reproducibility of these interventions in diverse patient populations. Second, the standardization of herbal formulations and variability in herbal quality, sourcing, preparation methods, and dosages contribute to inconsistent therapeutic outcomes. Therefore, establishing standardized guidelines for the production and clinical use of herbal medicines, including chemical profiling, pharmacokinetic analysis, and quality control measures, is urgently required. Third, there is mechanistic uncertainty and multi-target complexity, although several mechanisms of action have been proposed, ranging from anti-inflammatory and antioxidative effects to modulation of key signaling pathways such as PI3K/AKT, TGF-β/Smad, and NF-κB. The pleiotropic nature of herbal formulations complicates our understanding of their primary modes of action. Integrating modern tools, such as omics technologies, network pharmacology, and systems biology, may help unravel these complex mechanisms. Fourth, herb-drug interactions, safety concerns, and potential interactions between herbal components and conventional medications remain critical concerns, particularly in patients with advanced CKD who are often on polypharmacy regimens. Comprehensive pharmacovigilance and toxicity studies are required to ensure safe coadministration. Fifth, integration into conventional practice and bridging the gap between CHM and evidence-based Western medicine requires interdisciplinary collaboration, cross-cultural understanding, and the development of integrative care models. The education of healthcare providers and patients alike is vital for informed decision-making and for improving the acceptance of complementary therapies. Sixth, regulatory and policy barriers and differences in regulatory frameworks across countries pose additional barriers to the global adoption of CHM in nephrology. International consensus on evaluation criteria, quality standards, and approval pathways will facilitate broader access and research collaboration.

## 7. Critical Appraisal of Clinical Evidence

In evaluating the clinical evidence, several RCTs investigating CHM in the management of CKD and IgAN were critically appraised for methodological rigor. Most studies employed parallel-group designs with placebo or standard therapy controls, though variations in sample size, treatment duration, and herbal formulation standardization were observed. For example, trials assessing LW (21, 22) and SQW (29-32) included sample sizes ranging from 40 to 200 participants, typically conducted over 8 to 24 weeks, and demonstrated significant improvements in serum creatinine, eGFR, and proteinuria reduction compared with controls. However, some studies lacked blinding procedures, long-term follow-up, or detailed allocation concealment, which may introduce bias. To ensure balanced interpretation, we considered study design rigor, sample representativeness, and control measures in weighing the strength of evidence. Meta-analyses incorporating multiple RCTs further support the therapeutic benefits of CHM as an adjunct to conventional therapy but also emphasize the need for large-scale, multicenter trials with standardized protocols and quality control of herbal formulations (54). This critical appraisal underscores that while existing RCTs provide promising evidence, future studies should adopt more rigorous designs and comprehensive reporting standards to enhance clinical validity and reproducibility.

## 8. Safety Considerations and Risk-Benefit Evaluation

*Rheum officinale* (Da Huang) provides renal protection through the excretion of uremic toxins and reduction of fibrosis, but chronic or high-dose use may result in electrolyte imbalance (notably hypokalemia), gastrointestinal irritation, and potential oxalate nephropathy in susceptible patients (47-51). Most clinical studies have applied short-term (≤ 12 weeks) administration, which showed good tolerability, though long-term safety data remain insufficient. A comprehensive evaluation of the safety profiles and long-term effects of major Chinese herbal medicines (CHMs) was conducted to balance efficacy with potential risks. The TwHF demonstrates potent anti-inflammatory and immunosuppressive activities; however, its use is associated with dose-dependent hepatotoxicity, nephrotoxicity, reproductive toxicity, and gastrointestinal disturbances. Meta-analyses have reported a 10% incidence of mild adverse events, while serious toxicities are mainly linked to non-standardized extracts or high-dose regimens. Long-term safety data remains limited; therefore, clinical applications should prioritize standardized, quality-controlled preparations with close monitoring (52-57). Future investigations should incorporate systematic safety assessments, including dose-response studies, toxicological endpoints, and pharmacovigilance data, to guide safe integration of CHMs into CKD management and establish evidence-based safety guidelines.

## 9. Summary

This review explored the growing use of CHM and herbal medicine in the management of CKD and IgAN. This highlights how CHM approaches based on syndrome differentiation (e.g., kidney yin-yang deficiency, blood stasis, and dampness accumulation) offer multi-targeted mechanisms, including anti-inflammatory, antioxidant, anti-fibrotic, and immunomodulatory effects. Key traditional formulas, such as LW, ZWD, SQW, and YQW, have shown potential in improving renal function, reducing proteinuria, and slowing CKD progression in preclinical and clinical studies. Single herbs such as *A. membranaceus*, *S. miltiorrhiza*, *R. officinale*, *T. wilfordii* Hook F, and *C. sinensis* have demonstrated significant renoprotective properties, often through modulation of signaling pathways, such as PI3K/Akt, TGF-β/Smad, and NF-κB. Herein, we also discuss emerging evidence on CHM’s benefits in IgA nephropathy, including reduced proteinuria and inflammation, and its potential synergy with conventional treatments such as angiotensin-converting enzyme (ACE) inhibitors. Formulations, such as the Yi-Qi-Qing-Jie Formula and NPKL, are under investigation for high-risk patients with IgAN. Despite these promising results, challenges remain regarding standardization, clinical validation, safety profiling, and integration into mainstream nephrology. The authors called for high-quality RCTs, mechanistic studies, and regulatory harmonization to fully realize the potential of CHM in kidney disease care.

### 9.1. Conclusions

The CHM is a valuable but underutilized resource for CKD management. Accumulating preclinical and clinical evidence holds great promise for complementing conventional nephrology practices. Future research should prioritize rigorous clinical validation, pharmacological standardization, and integrative healthcare frameworks to unlock the full potential of CHM and improve outcomes in patients with kidney disease.

### 9.2. Limitations

While this review provides a comprehensive overview of CHM and herbal medicine in the treatment of CKD and IgAN, several limitations must be acknowledged. First, the heterogeneity in study designs, herbal formulations, dosages, and patient populations among the reviewed clinical and preclinical studies introduces variability that limits the generalizability of findings. Many of the clinical trials included were small-scale, lacked blinding or placebo control, and were conducted in single-center settings, which may introduce selection and publication bias. Second, the complexity and multi-component nature of CHM formulations present challenges in identifying specific active ingredients and their mechanisms of action. While network pharmacology and omics-based approaches have provided insight into potential pathways, definitive causal relationships remain to be fully elucidated. Third, standardization and quality control of herbal products remain inconsistent across different regions and manufacturers, affecting reproducibility and clinical reliability. Fourth, the potential for herb-drug interactions, particularly in patients with advanced CKD who are often on polypharmacy regimens, remains underexplored and warrants caution. Toxicological assessments and long-term safety data are limited for several frequently used herbs, such as *T. wilfordii* Hook F and *R. officinale*. Finally, despite promising preclinical and early clinical findings, there is a need for rigorously designed, multicenter RCTs with standardized protocols to validate the efficacy, safety, and mechanistic rationale of CHM interventions. Future research should also emphasize pharmacokinetics, regulatory harmonization, and integrative care models to enable the broader adoption of CHM in evidence-based nephrology.
